# Contribution quality evaluation of table tennis match by using TOPSIS-RSR method - an empirical study

**DOI:** 10.1186/s13102-023-00738-9

**Published:** 2023-10-13

**Authors:** Huagen Yin, Xia Chen, Yanxiang Zhou, Jiali Xu, Duo Huang

**Affiliations:** 1 School of Physical Education, Shangrao Normal University, Shangrao, 334001 Jiangxi China; 2 Graduate School, Adamson University, Manila, 1000 Philippines; 3 School of Physical Education, Putian University, Putian, 351100 Fujian China; 4 Shangrao Health Vocational College, Shangrao, 334600 Jiangxi China

**Keywords:** Table tennis, Technique for Order Preference by Similarity to Ideal Solution(TOPSIS), Rank Sum Ratio(RSR), Technique and tactic, Contribution quality evaluation

## Abstract

This paper aims to evaluate the contribution quality of table tennis matches comprehensively and explore the ranking characteristics of evaluation results and the rationality of grading. Through the application of the documentation method, videos, Technique for Order Preference by Similarity to Ideal Solution (TOPSIS) and Rank Sum Ratio (RSR), the contribution quality evaluation index system of table tennis matches was established. After then, the technical and tactical performances of 38 matches between H (anonymous), who is currently highly concerned and active in the international table tennis world from 2018 to 2020 were comprehensively evaluated. According to research results, H had 8 matches with the *C*_*i*_ value > 0.5 in serve rounds, 4 with the *C*_*i*_ value > 0.5 in receive rounds, and 5 with the RSR value > 0.6 in the comprehensive strength. These findings were generally consistent with the final match results. Furthermore, Pearson Correlation showed that the three indicators were significantly correlated with competition performance (CP) (*P* < 0.01). Each race could be divided into four grades, and there was a very significant difference among them by variance test (*F* = 60.281, *P* < 0.01). Meanwhile, SNK pairwise comparison between four grades had statistical significance (*P* < 0.05). Therefore, researchers could conclude that the combination of TOPSIS and RSR could objectively and accurately reflect the contribution quality of table tennis matches. This method could be promoted and applied in the competition performance evaluation of other net games.

## Introduction

As one of the sports that won the most titles in the previous world series, table tennis has prospered for nearly 60 years in China [1]. By analyzing various technical and tactical behaviors (such as disadvantages and advantages) of table tennis players in competitions through a large number of video analyses and mathematical analyses, scientific researchers could provide strategies for coaches to guide training and formulate competition strategies and tactics. Therefore, the diagnosis of techniques and tactics in table tennis matches can not only provide useful guidance for training, but also improve the competitive ability of athletes [2]. The initial application of the research method on table tennis techniques and tactics is the Three-Phase Evaluation Theory proposed by Wu et al. [3]. This method has laid the foundation for constructing the theoretical analysis system of table tennis techniques and tactics. Most current literature in this field has adopted this method, which has also been applied in other net sports [4]. Although this method has been proven to be intuitive and effective in practice, it still has some defects in that the technical data of both sides in matches do not correspond. Due to this advantage, many sports researchers have made a series of studies on the analytical theory of table tennis techniques and tactics to improve the evaluation and diagnosis method. For example, Li and Su [5] proposed Ten-Index Method, which sets the scoring rate, utilization rate and hit rate of serve, attack after serve, receive, attack after receive and rally are set as ten phase indexes. The application of this method effectively evaluates the technical strength of some world’s outstanding male offensive players. Li [6] put forward the concepts of contribution rate and rapid diagnosis of techniques and tactics in his master thesis, which provide faster and easier statistical processing of data, while solving the drawbacks of the traditional Three-Phase Evaluation Theory of double standards and difficult quantitative analysis. Zhang et al. [7] established the evaluation model of technical benefits by applying the quadratic function based on the relationship between the scoring rate and utilization rate. He set the attack after serve phase (the first and third stroke), attack after receive phase (the secnd and fourth stroke) and rally phase (after the fourth stroke) as the evaluation standard of technical and tactical benefits. Yang and Zhang [8, 9] constructed Four-Phase Evaluation Theory and Strength Difference Evaluation Method. The Four-Phase Evaluation Theory is to divide the original rally phase into rally I and rally II. The Strength Difference Evaluation Method is to quantitatively analyze the difference between the competitive ability of both players in each hitting phase, which overcomes the limitation of technical index evaluation. Jiang and Yao [10] proposed the Double-system Five-Phase Evaluation Method, in which the fifth and sixth strokes are listed separately by setting the serve and receive systems, and the other three indicators are similar to the Three-phase Indicators. Zhao and Tang [11] proposed the Technical and Tactical Level Evaluation Model for table tennis, that is, the corresponding evaluation indicators are selected from different levels of table tennis techniques and tactics, and the corresponding evaluation criteria are formulated through appropriate competition samples, which enriches the evaluation content to a certain extent. Yu and Gao [12] proposed the Interactive Three-Phase Structure for Table Tennis. It divides the rally competing process into three separate and cohesive phases: mutual restriction phase, initial attack and counterattack phase and topspin exchange phase). In addition, many researchers also depended on modern information technology and multi-disciplinary integration to study table tennis techniques and tactics. At present, the research methods involved in this project mainly include grey correlation theory (using gray correlation to establish the weight of table tennis technical indicators and constructing a multi-objective decision model) [13], log-linear modelling (describing the stroke and footwork types of players from different regions by analyzing the interrelationship of variables) [14], Deep Convolutional Neural Network-long Short Term Memory (DCNN-LSTM) model analysis (the model is used to identify and track the real-time trajectory of table tennis balls in complex environments, providing decisions for table tennis tactical evaluation) [15], Logistic Regression Model Analysis (by collecting the longitudinal data of table tennis match, the regression model of the first-three-stroke techniques and tactics of the players in the match is established) [16], Artificial Neural Network (ANN) (a three-layer BP neural network model for table tennis match diagnosis using the Dual Three-phase Index Evaluation Method as a framework can provide accurate prediction of match winning probability) [17, 18], Decision Tree (the decision tree model of table tennis match can be established by dividing the five phases of technical and tactical indicators, which can reasonably distinguish and evaluate the technical and tactical strength of both sides) [19], Particle Swarm (the particle swarm algorithm is used to model the evaluation indexes of table tennis special selection, and the separation and internal aggregation of various evaluation indexes can be derived) [20], etc. The intervention of these methods has had a certain effect on the study of table tennis techniques and tactics.

Throughout the study of scholars as mentioned above, many scholars conducted in-depth research on the evaluation system, models and data mining of table tennis techniques and tactics. Meanwhile, they have also found a better solution to the data mismatch caused by the attribution of the fifth-round score in the rally and the complex statistical (linear and non-linear) problems encountered in table tennis technical and tactical data mining [21]. However, the analysis of the technical and tactical data in each table tennis match is only treated as the independent individual in the current study, which lacks systematic evaluation and comparison of differences in the quality of games. Moreover, few comprehensive quantitative research results on table tennis techniques and tactics [22] and the lack of exploratory research on the ranking characteristics and comprehensive strength classification of table tennis four-phase (including each stroke) indicators are also limitations in this research field.

TOPSIS is a ranking method that approximates the ideal solution, and its basic idea is to transform the problem of comprehensive evaluation into finding the difference between each evaluation object, which is simple and easy to implement with few limitations [23]. RSR, namely Rank Sum Ratio, obtains dimensionless statistic RSR through rank transformation in a matrix with n rows and m columns, and ranks quality of evaluation objects according to RSR value [24]. Since the evaluation results of the TOPSIS are easily affected by outliers, the RSR can make up for and broaden the application scope of the TOPSIS. The complementarity of the two can effectively avoid the limitations of a single method and ensure that the evaluation results are more scientific and comprehensive [25]. At present, TOPSIS and RSR are widely used in many fields such as health decision-making and health management [26], and they also appear in the physical health evaluation in the field of sports, and the technical evaluation of football, basketball and volleyball games. However, most of them use a single evaluation method, and the combined application of TOPSIS and RSR in the field of sports is very few [27].

In view of this phenomenon, researchers tried introducing Technique for Order Preference by Similarity to Ideal Solution (TOPSIS) in combination with Rank Sum Ratio (RSR) [Cause: The combination of TOPSIS—RSR method can effectively avoid the limitations of using a single comprehensive evaluation method, achieve complementary advantages and make the evaluation results reliable.]. After reviewing 38 important matches between H, who is currently highly concerned and active in the international table tennis world, and key players from other states from 2018 to 2020, researchers could rank the technology and tactics contribution rate of each stroke and divide his comprehensive competitive strength of the single game in grades. Through comprehensive quantitative analysis, this paper studied the sorting characteristics and the rationality of gear division from an exploratory perspective to the competitive state characteristics of H. At the same time, it could provide references for table tennis enthusiasts to formulate scientific training plans and evaluate their technical and tactical abilities. This research also had practical significance for enriching the current theoretical system of technical and tactical application in table tennis and the practical guide of table tennis. Due to the few applications of comprehensive quantitative research in technical and tactical analysis, this study assumed that the combination of the eight sub-technical indicators constructed through the Four-phase indicators and TOPSIS—RSR method could comprehensively evaluate the contribution quality of various table tennis techniques and tactics, exploring the rationality of TOPSIS—RSR in ranking characteristics and gear division of table tennis techniques and tactics from a scientific perspective.

## Research objects and methods

### Research objects

This study took competitions between H, who is currently highly concerned and active in the international table tennis world, and main table tennis players worldwide as a case study. Meanwhile, the contribution quality of each stroke technique in 38 matches (including Asian Cup, All Japan Table Tennis Championship, World Table Tennis Champions, International Table Tennis Tour and World Cup) from 2018 to 2020 was the research object [Note: Contribution quality refers to the contribution rate of athletic performance in a certain sport (table tennis)]. He is right handed and loop with fast break is his main style. At present, he ranks top 10 in the world. All competition videos are from TV broadcasts on official websites and the Internet, and this study is not subject to approval by the local institutional ethics committee in accordance with national legislative regulations. Table 1 is the specific game video.

**Table 1:** The information about the 38 matches

Types of events	N	Year of tournaments	N	Win/Lose	Level of draws	N	Identification of players	N
Asian Cup	4	2018–2019	2–2	3–1	Others	4	H VS Chinese	22
ITTF Tour Open	27	2018–2020	13–8-6	15–12	1/8 finals	4	H VS European	6
Men’s world cup	5	2018–2020	1–2-2	2–3	1/4 finals	13	H VS Japanese	3
World Championships	1	2019	1	Lose	1/2 finals	10	H VS Korean	7
All Japan Championships	1	2020	1	Lose	finals	7		

## Research method

### Video observation

These 38 table tennis matches that H participated in from 2018 to 2020 were collected and downloaded through the official website of the International Table Tennis Federation (https://www.ittf.com), CCTV 5 + (https://sports.cctv.com), etc., and the video analysis was carried out. The score and loss of the last stroke technique and tactics at each point were counted.

The selection of video observation indicators was based on the four-phase indicators developed by Yang and Zhang [8]. According to the competition rules of table tennis and the logical relationship between the hitting rounds of table tennis, a table tennis match was divided into serve rounds and receive rounds. Compared with the three-phase indicator evaluation method, the four-phase indicator effectively resolved the data mismatch in analyzing the technique and tactic of the fifth beat with the three-phase indicator evaluation method [9]. In detail, serve rounds contain attack after serve (serve + the third stroke + the loss of the fifth stroke) and rally I phases (the score of the fifth stroke + the seventh stroke and later). Receive rounds contain attack after receive (receive + the fourth stroke) and rally II phases (the sixth stroke + the eighth stroke and later). In view of the adoption of the new ball (40 mm + plastic ball), due to the reduction of the ball speed and the increase of the match rounds [28], the technical and tactical system of table tennis has changed, from focusing on the-first three-strokes technique in the past to the comprehensive technical output, more and more rounds [29], and there are more technical and tactical indicators of table tennis competition. Selecting reasonable index plays an important role in evaluating the competitive level of athletes. In this study, the contribution quality evaluation system based on the sub-indicators of the Four-phase Indicator was designed to meet the needs of the times and to be objective, incorporating the effectiveness of scoring and utilization rates. Therefore, the data from video observation were counted with the score and the lost point of the last stroke as the final observation point.

In addition, in order to ensure the authenticity and reliability of the data, all the data collection was completed by us independently. We also trained three students majoring in table tennis to do the auxiliary observation records, and some of the data counted by the first author compared with the data counted by the three students passed the Kappa test with a Kappa value of 0.947, indicating good agreement of the observed data (Table 2).

**Table 2:** Kappa consistency test

Pairing Item	Kappa value	Standard error	*z*	*P*
Students & teachers	0.947	0.162	5.848	0.000^***^

^***^, ^**^ and ^*^ represent significance levels of 1%, 5% and 10% respectively

### Analysis of competition performance

The competition performance was first established by Zhang and Guan [30]. In table tennis matches with 11 points per game, the maximum score difference between both sides is 22, which means 11:0. When one player wins at 11:0 in each game, the competition performance (CP) of the winner is 22. The competition performance of the losing player is 0. The formula of calculation is as follows:

1 $$CP=22-\sqrt{\frac{\sum\limits_{i=1}^{n}{\left({x}_{i}-11\right)}^{2}}{n}}$$

*x*_*i*_ is the difference between scores of each game, *n* is the number of games.

### TOPSIS

C.L. Hwang and K. Yoonfirst proposed TOPSIS (Technique for Order Preference by Similarity to Ideal Solution) in 1981 [31], a common method for multi-objective decision analysis of effective schemes in systems engineering. This method aims to find the practical solution of the optimal scheme and the worst through the original data matric after normalization. Then the formula is applied to calculate the distance between evaluation objects and the optimal solution and between evaluation objects and the worst solution. Finally, the close degree of each evaluation object to the optimal scheme is obtained, which is also the basis of quality evaluation [22, 23].

The basic principle of TOPSIS is to find a solution among the feasible options that is closest to the ideal solution and farthest from the negative ideal solution based on the positive and negative ideal solutions of the decision problem. The ideal solution generally assumes the best solution, whose corresponding attributes are at least up to the best value among the options; the negative ideal solution is the assumed worst solution, whose corresponding attributes are at least not better than the worst value among the options. The rule of solution ranking is to compare the actual feasible solution with the ideal solution and the negative ideal solution. If a feasible solution is closest to the ideal solution and at the same time farthest from the negative ideal solution, then this solution is a satisfactory solution for the set of solutions [31, 32].

Step 1: The matrix data is normalized and processed with the following formula:

2 $${Z}_{ij}={X}_{ij}/\sqrt{\sum\limits_{i=1}^{n}{X}_{ij}^{2}}$$

In formula (2), *X*_ij_ means the i^th^ evaluation object on the j^th^ index, i = X1, X2, X3, …, X38; j = A1, A2, A3, …, A8.

Step 2: Determine the the optimal scheme A^+^ and the worst scheme A^−^

The normalized matrix A = (Zij)i × j is obtained, and the optimal and worst vectors composed of its maximum and minimum values are denoted as:

3 $$\mathrm{The\ optimal\ scheme\ } {A}^{+}=\left({a}_{i1}^{+},{a}_{i2}^{+},{a}_{i3}^{+},\cdots \cdots \cdots ,{a}_{im}^{+}\right)$$

4 $$\mathrm{The\ worst\ scheme\ } {A}^{-}=\left({a}_{i1}^{-},{a}_{i2}^{-},{a}_{i3}^{-},\cdots \cdots \cdots ,{a}_{im}^{-}\right)$$

In the above formula (3) and (4), ${a}_{ij}^{+}$ and ${a}_{ij}^{-}$ mean the maximum and minimum value of the j^th^ tactical index in each match. i = X1, X2, X3,…, X38; j = A1, A2, A3,…, A8.

Step 3: Calculate the distance, the Euclidean distance from the evaluated object to the positive ideal solution and the negative ideal solution.
  5 $${D}_{i}^{+}=\sqrt{\sum_{j=1}^{m}{\left({a}_{ij}^{+}-{a}_{ij}\right)}^{2}}$$

6 $${D}_{i}^{-}=\sqrt{\sum_{j=1}^{m}{\left({a}_{ij}^{-}-{a}_{ij}\right)}^{2}}$$

In the above formula (5) and (6), *D*_*i*_^+^ and *D*_*i*_^*−*^ mean the distance between the i^th^ evaluation index and the optimal scheme and the worst scheme and a_ij_ represents the value of some evaluation object (i) on the (j^th^) technical index [24]. i = X1,X2,X3,…,X38; j = A1,A2,A3,…,A8.

Step 4: Calculate the comprehensive evaluation value:

Calculate the relative proximity of each evaluation object to the optimal solution.

7 $${C}_{i}={D}_{i}^{-}/\left({D}_{i}^{-}+{D}_{i}^{+}\right)$$

In the above formula (7), the *C*_*i*_ value ranges from 0 to 1, which means 0 ≤ *C*_*i*_ ≤ 1. The closer the *C*_*i*_ value is to 1, the closer the evaluation object is to the optimal level. Conversely, the closer the Ci value is to 0, the closer the evaluation object is to the worst level [23]. I = X1, X2, X3,…, X38.

### RSR

Chinese statistician professor Tian Fengtiao first proposed RSR (Rank Sum Ratio) in 1988 [33], statistical method integrating the advantages of classical parameter estimation and modern non-parametric statistics. The basic idea of PRSR is the obtain the dimensionless statistic RSR by the rank transformation in a matrix with n rows and m columns. On this basis, the concept and method of parametric statistical analysis are applied to study the distribution of RSR. The RSR value is used to sort or classify the merits of evaluation objects. The formula of the RSR value is RSR = ∑R/(m × n). ∑R means the rank sum value of an evaluation object index, n is the evaluation object (The number of competitions), and m is the evaluation index (technical index). The value range of RAR is between (0–1). The larger the RSR value, the better it is. Otherwise, the smaller the RSR value, the worse it is [34]. According to continuous variables of the RSR, the five-grade evaluation scale of the RSR is selected, as shown in Table 3 (A is the superior grade, B is the medium to upper grade, C is the medium grade, D is the medium to lower grade, and E is the poor grade) [35]. A standard evaluation table was established to evaluate the competitive strength of H in 38 matches from 2018 to 2020.

**Table 3:** Comprehensive evaluation Table of RSR grade [33]

Evaluation grade	A-grade	B-grade	C-grade	D-grade	E-grade
Value range	≥ 0.80	0.60 ~ 0.79	0.40 ~ 0.59	0.20 ~ 0.39	≤ 0.19

### Mathematical statistics

Eight sub-technical indicators were determined by the four-phase indicators, including serve rounds (serve, the third stroke, the fifth stroke, the seventh stroke and later) and receive rounds (receive, the fourth stroke, the sixth stroke, the eighth stroke and later). For the convenience of statistics, eight indicators were set into corresponding codes (A1, A2, A3, A4, A5, A6, A7, A8). Microsoft Excel was applied to conduct modelling and statistics of the score and loss of each technical indicator at first. Then the scoring rate and utilization rate of each technical indicator were calculated according to the corresponding formula (Table 4), which was also applied to count the corresponding contribution rate of each indicator. Finally, the contribution rate of each technical index was imported into SPSS25.0 (SPSS Inc., Chicago, IL, USA) for data analysis.

**Table 4:** Contribution quality evaluation system of each stroke technique and tactics indicator in table tennis matches

Indicator	The formula of scoring rate		The formula of utilization rate	
Serve round	Serve	Serve points/Total serve points × 100%	Serve	Total serve points/Number of serve rounds × 100%
	The third stroke	Points in the third stroke/Total points in the third stroke × 100%	The third stroke	Total points in the third stroke/Number of serve rounds × 100%
	The fifth stroke	Points in the fifth stroke/Total points in the fifth stroke × 100%	The fifth stroke	Total points in the fifth stroke/Number of serve rounds × 100%
	The seventh stroke and later	Points in the seventh stroke/Total points in the seventh stroke × 100%	The seventh stroke and later	Total points in the seventh stroke/Number of serve rounds × 100%
Receive round	Receive	Receive points/Total receive points × 100%	Receive	Total Receive points/Number of receive rounds × 100%
	The fourth stroke	Points in the fourth stoke/Total points in the fourth stroke × 100%	The fourth stroke	Total points in the fourth stroke/Number of receive rounds × 100%
	The sixth stroke	Points in the sixth stoke/Total points in the sixth stroke × 100%	The sixth stroke	Total points in the sixth stroke/Number of receive rounds × 100%
	The eighth stroke and later	Points in the eighth stoke/Total points in the eighth stroke × 100%	The eighth stroke	Total points in the eighth stroke/Number of receive rounds × 100%
Contribution quality	Score rate × Utilization rate [6]			

## The empirical analysis

### Technical indicator TOPSIS evaluation of H in matches

#### Technical indicators and the same-trending and normalization matrix of each match

Before the original data matrix was established, the low-quality index was converted into the high-quality index by the reciprocal method (*X*_*ij*_^*’*^ = 100%/*X*_*ij*_) for same-trending, and the original data matrix after same-trending was established. Then, the normalization was carried out to eliminate the influence of different dimensions on the evaluation index. Tactical indicators (contribution rate) of each stroke selected in this study belong to high-performance indicators, so it is no need to conduct same-trending conversion, and normalization could be conducted directly. The specific treatment method was carried out according to formula (2). Table 5 shows the normalized matrix values of each stroke tactical indicator in 38 matches of H.

**Table 5:** Contribution quality normalization matrix of each Stroke technical and tactical index in matches between H and opponents

Event No	Serve round (attack after serve, rally I phases)				Receive round (attack after receive, rally II phases)			
	A1	A2	A3	A4	A5	A6	A7	A8
X1	0.1249	0.2248	0.2019	0.0472	0.0707	0.1401	0.3808	0.0805
X2	0.2087	0.2864	0.0000	0.2105	0.0960	0.2433	0.2067	0.1749
X3	0.2705	0.2262	0.1750	0.1535	0.1222	0.2258	0.1462	0.0927
X4	0.1660	0.1424	0.1074	0.0837	0.1250	0.0990	0.1122	0.0000
X5	0.1217	0.1629	0.2835	0.0737	0.1186	0.1461	0.0000	0.2400
X6	0.2480	0.1624	0.1167	0.1364	0.2371	0.0626	0.2364	0.0000
X7	0.1200	0.1059	0.0887	0.1816	0.2708	0.1430	0.1440	0.1370
X8	0.2682	0.1911	0.1335	0.1249	0.1414	0.1868	0.0846	0.0000
X9	0.0459	0.1300	0.2080	0.0695	0.1320	0.1331	0.1292	0.2186
X10	0.0716	0.0983	0.2162	0.3612	0.2150	0.1065	0.1929	0.1836
X11	0.0689	0.0946	0.2080	0.1738	0.1034	0.1228	0.1391	0.3531
·	·	·	·	·	·	·	·	·
··	··	··	··	··	··	··	··	··
···	···	···	···	···	···	···	···	···
X30	0.1667	0.1631	0.1079	0.0757	0.1565	0.1604	0.1321	0.2096
X31	0.0702	0.1205	0.1514	0.1771	0.1014	0.1808	0.1365	0.1732
X32	0.1469	0.1620	0.1358	0.1588	0.1158	0.2570	0.1247	0.2111
X33	0.1974	0.1524	0.0426	0.2489	0.2613	0.1479	0.2010	0.0850
X34	0.1660	0.1424	0.2148	0.2093	0.2813	0.0990	0.1122	0.0000
X35	0.1708	0.1319	0.1382	0.0969	0.1025	0.1624	0.2044	0.2075
X36	0.0206	0.1805	0.1602	0.2185	0.0672	0.2484	0.0804	0.2550
X37	0.0749	0.1638	0.1696	0.2267	0.0702	0.3019	0.0720	0.1370
X38	0.1679	0.1404	0.1901	0.0953	0.1025	0.1443	0.0817	0.1556

#### Determination of the optimal scheme ${A}^{+}$ and the worst scheme ${A}^{-}$ for each match

According to the data of the normalized matrix in Table 4, the optimal scheme *A*^+^ and the worst scheme *A*^*−*^ of the tactical indicators in each match were obtained through the optional value formula (3) and the worst value formula (4). Therefore, the vector sets of optimal scheme *A*^+^ and the worst scheme *A*^*−*^ of the tactical index in serve and receive rounds based on the normalized matrix and the formula of the optimal value and the worst value, as shown in below:

$$\begin{aligned}\text{Serve round}\ {A}^{+}&=\left(0.3043, 0.2864, 0.2835, 0.3612\right)\\ \text{Serve round}\ {A}^{-}&=\left(0.206, 0.0743, 0.0000, 0.0000\right)\\ \text{Receive round}\ {A}^{+}&=\left(0.2813, 0.3019, 0.3808, 0.3531\right)\\ \text{Receive round}\ {A}^{-}&=\left(0.0672, 0.0394, 0.0000, 0.0000\right)\end{aligned}$$

#### The calculation of the distance ${D}_{i}^{+}$ and ${D}_{i}^{-}$ between the technical index and the optimal scheme and the worst scheme for each match

According to the above formulas (5) and (6), the distance between the optimal scheme A^+^ and the worst scheme A^−^ for the technical indicators of H’s serve round and receive round in each match are calculated, as shown in Table 6.

**Table 6:** *C*_*i*_ and RSR distribution of the serve round and the receive round between H and his opponents

Event No	Serve round			*R*	Receive round			*R*	RSR	RSR rank/ grade
	*D* +	*D-*	*C*_*i*_		*D* +	*D-*	*C*_*i*_			
X1	0.3758	0.2766	0.4240	24	0.3806	0.4020	0.5137	4	0.5247	17/C
X2	0.3350	0.3531	0.5131	6	0.3160	0.3402	0.5184	3	0.6563	3/B
X3	0.2443	0.3738	0.6048	1	0.3924	0.2603	0.3988	21	0.6859	1/B
X4	0.3846	0.2105	0.3538	35	0.5123	0.1396	0.2141	38	0.3076	38/D
X5	0.3623	0.3223	0.4708	13	0.4567	0.2677	0.3695	25	0.4967	24/C
X6	0.3113	0.3028	0.4931	9	0.4525	0.2921	0.3923	23	0.5214	18/C
X7	0.3698	0.2274	0.3808	32	0.3580	0.3028	0.4582	14	0.5016	22/C
X8	0.2979	0.3292	0.5250	5	0.4952	0.1854	0.2724	35	0.5263	16/C
X9	0.4266	0.2277	0.3480	38	0.3636	0.2783	0.4336	16	0.4441	33/C
X10	0.3067	0.4247	0.5806	2	0.3265	0.3119	0.4886	10	0.5938	6/C
X11	0.3648	0.2761	0.4308	22	0.3495	0.3903	0.5276	2	0.4901	25/C
·	·	·	·	·	·	·	·	·	·	·
··	··	··	··	··	··	··	··	··	··	··
···	···	···	···	···	···	···	···	···	···	···
X30	0.3827	0.2159	0.3606	33	0.3435	0.2898	0.4576	15	0.5329	13/C
X31	0.3656	0.2427	0.3990	29	0.3729	0.2642	0.4147	18	0.4605	29/C
X32	0.3210	0.2594	0.4470	17	0.3393	0.3314	0.4941	7	0.5707	7/C
X33	0.3163	0.3180	0.5013	8	0.3582	0.3115	0.4651	13	0.6184	5/B
X34	0.2601	0.3402	0.5667	4	0.4879	0.2489	0.3378	28	0.5313	15/C
X35	0.3642	0.2332	0.3903	31	0.3221	0.3181	0.4969	5	0.5313	14/C
X36	0.3567	0.2910	0.4493	16	0.3855	0.3394	0.4682	12	0.5428	12/C
X37	0.3142	0.3019	0.4900	10	0.4320	0.3047	0.4136	19	0.5214	18/C
X38	0.3455	0.2670	0.4359	20	0.4304	0.2077	0.3256	30	0.4523	31/C

#### The calculation and sorting of the Closeness (C_i_) between the tactical indicator of the serve round and the receive round in each match and the optimal scheme

Table 6 shows the tactical indicator *C*_*i*_ value and ranking of serving rounds and receiving rounds in each match of H calculated according to formula (7).

From the serve round (Table 6), in the match between H and J (anonymous) in the group match (X3) of the 2018 China Open Tennis Tournament, the contribution quality of the serve round was the best, with the *C*_*i*_ value of 0.6048. Secondly, matches with excellent contribution quality of serve rounds were X10(0.5806), X27(0.5689), X34(0.5667), X8(0.5250), X2(0.5130), X15(0.5041), X33(0.5018), which indicated that H had excellent techniques and tactics in the serve round of the above matches. And it was consistent with the result of a big score lead in these matches. However, there were ten matches in which the *C*_*i*_ value of the serve round contribution quality ≤ 0.4, especially in X9(0.3480), X26(0.3506), X12(0.3534), and X4(0.3538). It could be found that H had poor score ability in the serve round of these matches. Except for the X12 (F anonymous), the *C*_*i*_ value of the other matches was consistent with the large margin of defeat. According to the Pearson correlation analysis between the serve round *C*_*i*_ value of H in each match and his competition performance (CP) in Table 7, the correlation coefficient, *r* = 0.515 (*P* < 0.01), shows a moderate correlation. This result indicates that the *C*_*i*_ value of the serve round can objectively reflect the technical and tactical performance of the serve round in each match of H. Furthermore, the *C*_*i*_ value of the serve round has a great influence on the CP.

**Table 7:** The correlation between the *C*_*i*_ value of the serve round, *C*_*i*_ value of receive round and RSR value in the match of H and Competition Performance (CP)

		CP	Serve round *C*_*i*_ value	Receive round *C*_*i*_ Value	RSR value
Competition Performance (CP)	Pearson correlation	1	0.515^**^	0.512^**^	0.851^**^
	*P* value		0.001	0.001	0.000
	N	38	38	38	38

^**^P < 0.01

From receive rounds (Table 6), four matches of X22(0.5914), X11(0.5276), X2(0.5276), X1(0.5137) with *C*_*i*_ value > 0.5 belonged to the matches with good receive round techniques and tactics, which was coefficient with results of matches. Only X11 (vs K anonymous), the *C*_*i*_ value of the receive round was contrary to the result of the match. On the one hand, H could not perform his techniques and tactics in this match, which led to the decline of his comprehensive strength. On the other hand, he could not score through his advantages, making it difficult to make up for his shortcomings. Moreover, 16 matches with 0.4 ≤ *C*_*i*_ value < 0.5 belonged to the average technical and tactical performance of the receive rounds, and 18 matches with *C*_*i*_ value < 0.4, especially five matches including X25(0.2735), X8(0.2724), X20(0.2448), X28(0.2411), X4(0.2141) with *C*_*i*_ value < 0.3, indicating that the technical and tactical performance of the receive round was fragile. According to the Pearson correlation analysis between the receive round *C*_*i*_ value in each match of H and his CP in Table 7, the correlation coefficient r was 0.512 (*P* < 0.01), showing a moderate correlation. The result indicated that the receive round *C*_*i*_ value could objectively reflect the technical and tactical performance of the receive round in each match of H. Moreover, the receive round *C*_*i*_ value had a great influence on the CP.

## The application of RSR in ranking and grading of comprehensive strength in each match of H

### Ranking of comprehensive strength in each match of H

This paper applied the RSR value to evaluate and analyze the comprehensive competitive strength of H in each match. According to statistical results of RSR value, ranking and grade in Table 6, these 38 matches of H from 2018 to 2020 could be divided into three grades. Compared with other matches, X3(0.6859), X15(0.6595), X2(0.6563), X22(0.6332) and X33(0.6184) had the strongest comprehensive strength. In the meantime, the RSR value > 0.6, belonging to the B level (above medium), was consistent with the winning result. 30 matches with the RSR value ranging from 0.5938 to 0.4227 had slightly better comprehensive strength, belonging to C (medium) level. Only 3 matches with RSR reaching grade D (lower than medium), X20(0.3766), X28(0.3602) and X4(0.3076), which were consistent with the results of losing the match. From the above results, the comprehensive competitive strength in 38 matches H participated in from 2018 to 2020 was at a medium grade and few matches with particularly outstanding or weak comprehensive competitive strength. According to the Pearson correlation analysis between the RSR value in each match and CP in Table 7, the correlation coefficient, *r* = 0.851 (*P* < 0.01), shows a high correlation, which means that the RSR value could objectively and accurately reflect his comprehensive competitive strength in each match. Meanwhile, the overall strength RSR value had the greatest impact on the CP.

### Determination of comprehensive strength RSR value distribution

The distribution of RSR was the specific downward cumulative frequency of RSR values expressed by the Probit. The specific operation was as follows:

1. Researchers compiled the distribution table according to the order of RSR value from small to large and listed the frequency (f) among groups to calculate the cumulative frequency (Σf) among groups and determine the rank (R) and average rank (R) of RSR among groups;
2. Researchers calculated the downward cumulative frequency (P) = (R/n) × 100% and then converted the percentage (P) into the corresponding Probit, whose Probit was the standard-normal-deviate (u) plus 5 corresponding to the percentage (P) [24]. The converted result is shown in Table 8;
3. The regression equation was calculated by RSR = a + b × Probit, taking the Probit value of the cumulative variable as the independent variable and the RSR value as the dependent variable.

**Table 8:** RSR value distribution of H’s comprehensive strength in matches with his opponents

RSR distribution value	f	Σf	R	$\overline{R }$	$\left(\overline{\mathrm{R} }/\mathrm{n}\right)$*100%	Probit
0.3076	1	1	1	1	2.6	3.062
0.3602	1	2	2	2	5.3	3.380
0.3766	1	3	3	3	7.9	3.588
0.4227	1	4	4	4	10.5	3.748
0.4293	1	5	5	5	13.2	3.881
0.4441	1	6	6	6	15.8	3.997
0.4457	1	7	7	7	18.4	4.101
0.4523	1	8	8	8	21.1	4.195
0.4539	1	9	9	9	23.7	4.284
0.4605	1	10	10	10	26.3	4.366
0.4737	1	11	11	11	28.9	4.445
0.4885	1	12	12	12	31.6	4.520
0.4901	2	14	13, 14	13.5	35.5	4.629
0.4967	1	15	15	15	39.5	4.733
0.5000	1	16	16	16	42.1	4.801
0.5016	1	17	17	17	44.7	4.868
0.5049	1	18	18	18	47.4	4.934
0.5164	1	19	19	19	50	5.000
0.5214	2	21	20, 21	20.5	53.9	5.099
0.5247	1	22	22	22	57.9	5.199
0.5263	1	23	23	23	60.5	5.267
0.5296	1	24	24	24	63.2	5.336
0.5313	1	25	25	25	65.8	5.407
0.5329	1	26	26	26	68.4	5.480
0.5428	1	27	27	27	71.1	5.555
0.5510	2	29	28, 29	28.5	75	5.674
0.5641	1	30	30	30	78.9	5.805
0.5707	2	32	31, 32	31.5	82.9	5.950
0.5938	1	33	33	33	86.8	6.119
0.6184	1	34	34	34	89.5	6.252
0.6332	1	35	35	35	92.1	6.412
0.6563	1	36	36	36	94.7	6.620
0.6595	1	37	37	37	97.4	6.938
0.6859	1	38	38	38	99.3	7.479

Tables are estimated as (1–1/4*n)

In this case, the multivariate correlation coefficient (r) was 0.987, the determination coefficient (r^2^) was 0.974, and the adjusted r^2^ was 0.973, indicating that the whole set of regression equations could explain the difference of RSR distribution values to a high degree. ANOVA was *F* = 1178.410, *P* = 0.000 < 0.01(RSR = 0.107 + 0.08 × Probit), indicating that the linear regression equation had statistical significance (Results shown in Table 9).

**Table 9:** Linear regression model

	Unstandardized Coefficients		Standardized Coefficients	*t*	*p*	*r*	*r*^*2*^	Adjusted r^2^	*F*
	B	SE	Beta						
Constant	0.107	0.012	-	8.937	0.000	0.987	0.974	0.973	*F* (1,32) = 1178.410, *P* = 0.000
Probit value	0.08	0.002	0.987	34.328	0.000				

Dependent variable: RSR distribution value

## Determination of grading and ranking

According to the best grading standard of RSR and the table of reasonable grades [35, 36], the contribution quality of these 38 matches would be graded into four ranks, including excellent, good, average and poor. X3(0.707), X15(0.664) and X2(0.638) of the first rank had the best comprehensive strength. The RSR critical value of 16 matches with great comprehensive strength in the second rank ranged from 0.516 to 0.621. In the third rank, the RSR critical value of 17 matches with average comprehensive strength ranging from 0.395 to 0.508. Finally, two matches with the lowest comprehensive strength in the fourth rank were X28(0.378) and X4(0.353), as shown in Table 10.

**Table 10:** Grading and ranking of comprehensive strength in each match of H

Rank	Px	Probit	RSR critical value	Grade	Ranking and grading results	Number of matches
First	93.319 ~	7 ~	0.628 ~	Excellent	X3(0.707);X15(0.664);X2(0.638)	3
Second	50.000 ~	5 ~	0.508 ~	Good	X22(0.621);X33(0.609);X10(0.598);X32(0.584); X17(0.584);X27(0.573);X16(0.562);X24(0.562); X36(0.553);X30(0.547);X35(0.541);X34(0.535); X8(0.530);X1(0.524);X6(0.516);X37(0.516)	16
Third	6.681 ~	4 ~	0.388 ~	Average	X21(0.508);X12(0.503);X7(0.498);X19(0.492); X5(0.487);X11(0.478);X29(0.478);X23(0.470); X13(0.464);X31(0.457);X26(0.451);X38(0.444); X25(0.436);X9(0.428);X18(0.418);X14(0.408); X20(0.395)	17
Fourth	< 6.681	< 4	< 0.388	Poor	X28(0.378);X4(0.353)	2

After four grades determined by contribution quality to 38 matches in Table 10 were tested for homogeneity of variance by Levene analysis, Levene statistic = 0.506, *P* = 0.681 > 0.05, which showed the high variance consistency of all grades and met the prerequisite conditions of variance test. The results of the variance test showed that *F* = 60.281, *P* < 0.01, indicating that the difference between grades had statistical significance. Moreover, the Student–Newman–Keuls (SNK) pairwise comparison showed that the classification was effective because differences among four grades were statistically significant (*P* < 0.05 (Table 11 is the results).

**Table 11:** One-way analysis of variance for RSR critical values

Range	Square sum	df	Mean square	*F*	*P*	Homogeneity of variance test	
						Levene statistics	*P*
Interblock	0.2	3	0.067	60.281	0.000	0.506	0.681
Interclass	0.038	34	0.001				
Sum	0.238	37					

## Discussion and analysis

### Results of the comprehensive strength ranking and grading

According to the ranking and grading results of 38 matches that H participated in from 2018 to 2020 in Table 10, there were only three matches between him and the player with the strongest comprehensive strength, which was consistent with the results of matches won by a large score. However, based on the *C*_*i*_ value analysis of the serve round and the receive round, the match (X3) is in the grade with the strongest comprehensive strength, and the first rank has the highest value in the serve round, consistent with the comprehensive strength ranking. The *C*_*i*_ value ranking of the receive round was 21^st^, which was at the lower average grade, and quite different from the ranking result of the comprehensive strength in the match. At the technical level, in the X3, H had excellent techniques and tactics in the serve round, especially the high contribution quality of attack after serve. Meanwhile, H could maintain a high-pressure situation and active attack with fewer mistakes from the attack after serving to stalemate phases. However, his techniques and tactics of the receive round were average. As a result, the contribution quality of serve rounds and receive rounds in this match showed a bipolar trend. This phenomenon illustrated that his superior techniques could make up for the mediocre or weak techniques in the match, so the overall strength of X3 was better than that of other matches. In previous studies, Chen [37] and Yin [38] have clearly pointed out that Chinese table tennis players Liu Shiwen and Ding Ning have experienced the phenomenon of unbalanced competitive strength with too obvious good and poor techniques in their matches, which is similar to the view that exists in this study. In terms of evaluation methods, the combination of TOPSIS and RSR contributes to the objectivity and accuracy of the comprehensive strength in each match so that the comprehensive strength of X3 could clearly distinguish the gap with other matches. Otherwise, researchers further analyzed the ranking and grade of the following groups, including X36 (3:4) and X30 (3:4) in the second grade as well as X7 (4:3), X19 (4:1) and X5 (4:2) in the third grade. Theoretically, the overall strength of the winning rounds in the third grade should be in a higher grade, especially since H won by a large score in X19. By contrast, the overall strength of the losing matches in the second grade should have been lowered, but the overall strength of the winning race in the third grade was higher than the winning race, which was quite different from the expectation of the theoretical and actual results. However, researchers had new findings through game videos and the above analysis. The chance of winning or losing a table tennis match has increased since the development of the 11-point system in table tennis and the implementation of the new material table tennis. In the meantime, winning or losing at a high level is decided by the most critical points. The imbalance of the winning and losing relationship in the above matches in this study is consistent with the problems raised by Huang [39] and Cui [40] in their research results. There is a 5% probability of total score-loss imbalance (i.e., a player wins the match but scores less than his opponent) occurred in international male table tennis match. Therefore, the individual technical and tactical indicators of table tennis players can be applied to objectively reflect the effect of technical and tactical play in each stage by selecting the contribution quality of individual technical and tactical indicators and using the comprehensive evaluation combining TOPSIS and RSR. This method could conduct a more objective and comprehensive evaluation of the overall strength of a match. Prior to this, Yang et al. [27] conducted a comprehensive evaluation of the attack and defense ability of volleyball players in the competition by combining TOPSIS and RSR method, and believed that the combination of the two could comprehensively evaluate the attack and defense strength of each team, as well as the ranking of guard positions, which had certain reliability and rationality. In his study, Zhao and Tang [32] used TOPSIS alone to evaluate the competition quality of two high-level Chinese table tennis players, and the comprehensive ranking could also reflect the competitive status of the players to a certain extent. It shows that the combined application of the two comprehensive evaluation methods is feasible to diagnose the contribution efficiency of table tennis matches. In this regard, athletes can understand their technical and tactical deficiencies through comprehensive evaluation and analysis. Meanwhile, the analysis of their advantages and disadvantages in techniques and tactics when competing with strong and weak players could help athletes carry out targeted training for athletes to strengthen their weak techniques in future training. In this way, their techniques can provide stable and changeable intentions for implementing tactics in field competitions. Furthermore, coaches can help athletes to formulate corresponding tactical training based on analytical results. Afterwards, athletes could further understand their shortcomings in field competitions to strengthen the connection and conversion of techniques and tactics in the future and avoid polarized performances (the technical and tactical play is volatile) [37–40].

### The selection of various evaluation indicators

Table tennis matches have diverse evaluation indexes, such as the initial three-phase index, ten-phase index, and more widely used four-phase index. All of these methods aim to conduct statistics on the score and loss of each technique and tactic. However, some scholars analyzed the use of the active attack, spin serve, control, defence, position, hit placement and other indexes to study the technique and tactics of table tennis. Some scholars directly analyzed the scoring effect or losing effect of technique and tactic in each stroke. For example, unilateral evaluation of the scoring rate of various indicators in table tennis could not objectively evaluate the comprehensive competitive strength of athletes because the loss of points in the competition was ignored, leading to different evaluation results. Moreover, the evaluation composed of technical and tactical indicators such as an attack, defence, control, and position involves too many technical and tactical indicators (e.g.: according to the characteristics of the athlete’s position, there are short court attack after receive, middle court or back court counterattack, rally or defense, etc.). In the meantime, it was difficult to collect technical and tactical data. The implementation effect of technical and tactical could only be obtained from the unilateral score or loss, so it was laborious to highlight the contribution quality of table tennis matches. According to the previous table tennis technical phase can be divided into attack after serve phase, attack after receive phase and rally phase.With the reform of table tennis rules and equipment, the past Three-phase table tennis technology has been unable to meet the needs of current table tennis technology statistics, and there is also the problem of table tennis competition data statistics not corresponding [8]. Therefore, in terms of the selection of technical indicators in table tennis matches, Zhao and Tang used TOPSIS to evaluate the scoring rate of six indicators, including serve, attack after serve or control (the third stroke), receive, continuous attack after receive or control (the fourth stroke) and rally technique [32]. When Wang used RSR to analyze the offensive techniques of women’s table tennis matches, he selected the hit rate and scoring rate of serve, attack after serve, attack on the fifth stroke and attack after the seventh stroke as indicators to evaluate the offensive techniques of athletes [41]. These studies are sub-indicators selected on the basis of Three-phase technical indicators, which fail to consider the problems corresponding to the competition data and the utilization rate of athletes. In the match, the athletic performance of athletes cannot be reflected only by the scoring rate, which is not comprehensive enough. Each point scored or lost in the match needs to be converted into a scoring rate and utilization rate to determine the effect of the athlete’s technical efficiency output. High scoring rate and low utilization rate or high utilization rate and low scoring rate reflect the technique level of athletes. The contribution rate includes the effect of scoring rate and utilization rate, and the contribution rate of athletes in the corresponding phase can directly reflect the quality of athletes’ contribution per stroke. Therefore, based on previous studies, this study selects the four-phase index (Purpose: the four-phase index effectively solves the problem that the data of the fifth stroke was not corresponding), including the serve round------the attack after serve (the first stroke, the third stroke, the loss of the fifth stroke) and the stalemate I phase (the score of the fifth stroke, the seventh stroke and later), the receive round------the attack after receive (the first stroke, the third stroke, the loss of the fifth stroke) and the stalemate II phase (the sixth stroke, the eighth stroke and later) and the score and loss of the last stroke as statistical points. The scoring rate and utilization rate were calculated by the score and loss in each stroke. Through this way, researchers could obtain the contribution quality of each stroke. This index makes up for the shortcoming that some scholars only analyze the competition quality from the score but ignore the utilization effect of techniques in matches. Meanwhile, as an easy and understandable evaluation method, the contribution quality of each stroke in the four-phase index can objectively and comprehensively reflect the actual differences between single or multiple matches, which makes the evaluation results more representative than other methods. It can also provide decision-making guidance for coaches to clearly understand the contribution effect of athletes in a certain technical phase or a certain stroke in the match. In addition, this study focused on applying TOPSIS and RSR in the comprehensive evaluation of the contribution quality of techniques and tactics in table tennis matches, aiming to provide a new method and idea for analyzing techniques and tactics. In evaluating technical and tactical indicators based on different evaluation purposes in the specific operation process, the evaluation indicators could be adjusted according to the corresponding evaluation purposes. In the meantime, the evaluation could be added when athletes could implement other corresponding technical and tactical indicators in the competition, which was more representative of evaluating the comprehensive competitive strength of athletes.

### The application of the evaluation method

TOPSIS and RSR are two frequently-used comprehensive evaluation methods without special requirements for the data used. Currently, the relatively widely applied fields of TOPSIS mainly focus on enterprise performance management, health decision-making and public health management, etc. [41, 42]. In sports, they were also applied to evaluate the competition performance of basketball, football and volleyball [27, 34, 36]. RSR is more used in basketball. The main advantages of the two comprehensive evaluation methods are simple operation, flexible application, objective and accurate measurement of the evaluated objects, and there are no special requirements on the size of the sample, the number of evaluation objects and the distribution of index data. For example, the same trend transformation and normalization of the raw data by TOPSIS can eliminate the influence of different index levels, and the ranking results make full use of the raw data information, which can quantitatively reflect the degree of superiority and inferiority of different evaluation stages, and have certain practical value in the evaluation of contribution quality indexes of table tennis tournaments. Moreover, the resulting data processing results are easy to understand and more in line with the actual situation of table tennis match. However, when a particular index has a significant degree of dispersion, the results calculated by TOPSIS may not be stable, and the advantages and disadvantages of evaluation objects cannot be classified [41]. Due to this, RSR can cover the shortcomings of TOPSIS and broaden the application range of TOPSIS. On the other hand, TOPSIS can fill the fault of RSR, which is resulted from excessive information loss due to non-parametric transformation. The combined application of both methods can carry out reasonable evaluation and classification, which improves the statistical efficiency and makes the evaluation results more objective by complementing both advantages [43], avoiding the limitations of a single evaluation method. According to the previous literature, in the field of sports, whether it is Chinese literature or foreign literature, it is common to use a single method (TOPSIS or RSR) for quality evaluation, and to some extent there is unreasonable index evaluation phenomenon. However, in the field of public health, there are many literatures that use TOPSIS combined with RSR for comprehensive evaluation. For example, TOPSIS is used for comprehensive evaluation of hospital medical quality, while RSR is used for more reasonable classification evaluation based on TOPSIS analysis. Therefore, the combination of the two can achieve complementary advantages and avoid unreasonable single evaluation [32]. In addition, by comparing the comprehensive evaluation of the four-phase indicators on the competitive performance of each match, it is found that the four-phase indicator evaluation can separately assess the competitive strength of each phase of each game. For example, according to Yang and Zhang’s “four-phase index evaluation method” and “four-phase index strength difference method”, the scoring rate, utilization rate and strength difference of four-phase indexes are divided into different evaluation levels based on the scoring rate and utilization rate [8, 9]. In terms of the contribution rate of four-phase indexes, the diagnostic formula of four-phase indexes’ contribution rate extended by Yin et al. [44]. can effectively diagnose the magnitude and advantages and disadvantages of the contribution rate of each phase index in each match. However, the four-phase indicator evaluation method mentioned above only evaluates the competitive performance of each phase of each match, and cannot assess, rank and archive the comprehensive strength of each match. Therefore, TOPSIS combined with RSR method for table tennis competitive strength evaluation can effectively optimize the above existing defects. Based on this consideration, this study combines two methods. This combination changed the traditional evaluation methods adopted in previous studies of table tennis techniques and tactics to avoid the shortcomings such as complicated index selection, sophisticated calculation, and dispersed evaluation. Meanwhile, it could enhance objectivity, rationality and accuracy in the comprehensive strength evaluation in table tennis matches. So it can provide scientific evidence for the training of athletes and the decisions of coaches. Meanwhile, this method is also worthy of further promotion and application in net games.

### The limitations of this study

There were still some limitations in this study. First, this study was only evaluated unilaterally from the match data of H, a International Excellent table tennis player. It was impossible to directly and objectively infer the competitive state of the other player in the match. So, data from both athletes could be included for comparative evaluation and analysis in future studies. Second, due to the impact of the epidemic, many important international table tennis matches were suspended, which led to the imbalance between the selection of different matches and the designated time period, failing to achieve real-time tracking and statistics. In addition, the grib method and technical characteristics of the opponent are not specifically described in the paper, which leads to the limited application value of this study to a certain extent. It is hoped that relevant scholars can further improve the design and analysis of the comprehensive evaluation of competitive strength in table tennis match in the future. Finally, this study only quantified the game data from videos and ignored the psychological changes of the athletes in the game. In some critical games, the loss or win was not a technical or tactical problem but a psychological problem. For example, an athlete usually showed more flexible and steady techniques and tactics when he was ahead by a large margin. Due to this variable, future studies should pay attention to the combination of quantitative research on the technical and tactical index data of athletes and qualitative research on clinical performances to analyze techniques and tactics.

## Conclusion

1. The contribution quality evaluation of the serve and receive rounds in 38 matches of H were consistent with the match results, indicating that TOPSIS evaluation could reflect the competitive performance of H when he competed with players at different levels in serve and receive rounds. Moreover, the contribution quality evaluation of the eight sub-technical indicators constructed through the “four-stage index” was important for evaluating the overall competitive strength of table tennis players.
2. Based on the distribution characteristics of the RSR values, the combined strength of H in the 38 matches from 2018 to 2020 could be divided into 4 grades. In the meantime, the differences between all 4 grades were statistically significant (*P* < 0.05), indicating that RSR method could reasonably divide the 38 important matches of H.
3. The combination of TOPSIS and RSR could complement each other to avoid the limitations of a single evaluation method. In the application of this study, this combined method could objectively and accurately reflect the contribution quality of table tennis matches. Due to its reliability and reference for evaluating the technical and tactical play of table tennis players, it could be promoted and applied to evaluating the match effects of net games.

## Data Availability

The datasets used and/or analysed during the current study are available from the corresponding author on reasonable request.
